# Effect of Ion Irradiation on Nanoindentation Fracture and Deformation in Silicon Carbide

**DOI:** 10.1007/s11837-021-04636-8

**Published:** 2021-05-07

**Authors:** Alexander J. Leide, Richard I. Todd, David E. J. Armstrong

**Affiliations:** 1 Department of Materials, University of Oxford, Parks Road, Oxford, OX1 3PH UK; 2 Present Address: School of Physics, Tyndall Avenue, Bristol, BS8 1TL UK

## Abstract

Silicon carbide is desirable for many nuclear applications, making it necessary to understand how it deforms after irradiation. Ion implantation combined with nanoindentation is commonly used to measure radiation-induced changes to mechanical properties; hardness and modulus can be calculated from load–displacement curves, and fracture toughness can be estimated from surface crack lengths. Further insight into indentation deformation and fracture is required to understand the observed changes to mechanical properties caused by irradiation. This paper investigates indentation deformation using high-resolution electron backscatter diffraction (HR-EBSD) and Raman spectroscopy. Significant differences exist after irradiation: fracture is suppressed by swelling-induced compressive residual stresses, and the plastically deformed region extends further from the indentation. During focused ion beam cross-sectioning, indentation cracks grow, and residual stresses are modified. The results clarify the mechanisms responsible for the modification of apparent hardness and apparent indentation toughness values caused by the compressive residual stresses in ion-implanted specimens.

**Supplementary Information:**

The online version contains supplementary material available at 10.1007/s11837-021-04636-8.

## Introduction

Silicon carbide is a high-performance engineering ceramic useful for many nuclear environments including the blanket of fusion reactors, accident-tolerant fuel cladding in fission reactors, and as a component of tri-structural isotropic (TRISO) nuclear fuel particles.^1^–^3^ It is vital to understand the evolution of material properties with radiation damage to ensure safe, high-performance operation of nuclear reactors. To accelerate this research, ion irradiation is commonly used to introduce displacement damage to materials, and mechanical properties can be extracted using methods such as nanoindentation.^4^–^6^

Several researchers have observed larger radiation-induced hardening in ion-irradiated SiC^7^–^13^ compared with neutron-irradiated SiC,^7^,^14^–^16^ and this has recently been partially attributed to compressive residual stresses caused by constrained swelling of the irradiated layer.^7^,^13^ Changes to fracture, and plastic and elastic deformation have not been investigated in detail, which this paper aims to address.

The effect of irradiation on nanoindentation deformation mechanisms and the reasons for the large changes to nanoindentation hardness have not been investigated sufficiently in SiC, however differences have been observed in metals such as tungsten. Elastic and plastic deformation around nanoindents in unirradiated and helium- or self-ion-irradiated tungsten has recently been investigated using high-angular-resolution electron backscatter diffraction (HR-EBSD), Laue diffraction, and crystal plasticity finite element modeling (CP-FEM), finding localization of dislocations and elastic strain near indent impressions after irradiation, and different pile-up characteristics.^17^,^18^ To accurately replicate experimental results, the authors included biaxial compressive stress in the CP-FEM model to represent constrained lattice swelling, while they attribute most of the hardening and deformation changes to radiation defects.

Fracture toughness is an important property to measure in brittle materials to help design against sudden catastrophic failure. Since the original Griffith description of rupture in elastic solids, experimental techniques have been developed to measure this.^19^ Fracture toughness can be estimated using indentation combined with semi-empirical equations by measuring surface crack lengths and indentation load. The empirical equation is based on the indenter geometry and an assumed subsurface crack morphology.^20^–^22^

Jiang et al. reported on nanoindentation fracture toughness of 4H-SiC after xenon ion implantation at room temperature, finding that fracture toughness apparently increases with dose and in proportion to out-of-plane elastic strain caused by radiation swelling, up to ~75% apparent toughening.^23^ They attribute the crack shortening to lateral compressive stress in the irradiated layer. However, the equation used for the toughness measurements is not given, and the physically different effects of crack shortening by residual stress and fracture toughness are not clearly separated. Nogami et al. report on indentation fracture toughness of nanocrystalline 3C CVD SiC after neutron irradiation, finding a decrease of ~10% below 400°C and an increase above ~800°C, although with large scatter, possibly due to microstructure effects.^15^ The high load indentations in Ref. 15 are assumed to cause semicircular cracks, whereas low-load nanoindentations are expected to cause Palmqvist cracks, therefore requiring different fracture toughness calculations.^23^,^24^ The different conclusions from ion irradiation and neutron irradiation on apparent indentation fracture toughness require deeper investigation, especially in the context of using ion implantation as a surrogate for neutron irradiation.

To investigate indentation fracture and deformation in more detail, the subsurface features of indents have traditionally been investigated by sequential mechanical polishing and imaging, either from the top surface^25^ or from the side.^26^ This is labor intensive and imprecise due to the polishing step sizes, and only works well for larger indents. As the sectioning and imaging take place in different machines, dynamic changes cannot easily be seen.

Focused ion beam scanning electron microscopy (FIB-SEM) allows nanoscale milling with the FIB to reveal subsurface features of materials for imaging with the SEM column.^27^,^28^ This technique has been used to investigate subsurface indentation fracture in alumina,^29^ silicate glass,^30^ SiAlON,^31^ and silicon nitride.^32^ Local residual stresses were observed to cause bulging of FIB cross-sections, and the direction of FIB milling through the indent changed the observed crack density as residual stresses were relieved.^29^, ^30^

This work aims to explore the relationships between fracture, plastic deformation, and residual stresses around nanoindentations in unirradiated and irradiated silicon carbide, and how this affects mechanical properties measured by nanoindentation. FIB cross-sectioning is applied to investigate subsurface fracture. HR-EBSD analysis is used to map the elastic strain tensor and dislocation densities on the scale of the indentations in order to develop a better understanding of these relationships.

## Methods

A specimen of 6H-SiC single crystal, with surfaces parallel to the (0001) basal plane, was purchased from Pi-Kem Ltd (Tamworth, UK) in a pre-polished condition. No residual stress was observed in the as-received specimens based on Raman spectroscopy. This sample was irradiated with neon ions at 300°C using three ion energies of 1450 keV, 720 keV, and 350 keV to fluences of 9.4 × 10^15^ ions/cm^2^, 6.1 × 10^15^ ions/cm^2^, and 3.71 × 10^15^ ions/cm^2^ using beam currents of 40 nA, 50 nA, and 60 nA, respectively. The beam was scanned in a pseudo-random pattern to create the target dose uniformly across the samples while minimizing synergistic effects of a regular raster scan. This gave a flattened damage profile with a peak nominal damage ~2.5 dpa between 0.4 µm and 1.2 µm. A section of the same specimen was blanked from the ion beam to provide an unirradiated reference region which had been exposed to the same temperature cycle. Further details of the irradiation can be found in Ref. 13. These ion irradiation conditions cause a compressive residual biaxial stress of several GPa in the damaged layer.^13^

Nanoindentation was performed using an MTS Nanoindenter XP with a diamond Berkovich tip. The tip shape and frame compliance were calibrated before each batch of indents using a fused silica standard sample. Mechanical properties were calculated as a function of indentation depth using the continuous stiffness method (CSM). The CSM harmonic displacement was 2 nm with a frequency of 45 Hz and a strain rate of 0.05 s^−1^. The crystallographic orientation of the sample was kept constant with respect to the Berkovich tip while indenting the blanked and unblanked regions of the sample. Hardness increased by 12% to 15% between 400 nm and 1 µm indentation depth, approximately corresponding to the region of peak radiation damage. Elastic modulus was reduced by between −6% and −2% over the same depth, varying as a function of indentation depth.^13^ As elastic modulus is a long-range effect, at deep indentation depths, the measured modulus returns to the unirradiated value as the undamaged substrate makes a larger contribution to the measurement. Representative 1-µm-deep indents were selected for analysis in more detail in this work.

Fracture toughness was calculated using the modified Laugier equation^33^ from Dukino and Swain:^34^,^35^

1 $$ K_{c} = x_{v} \left( \frac{a}{l} \right)^{{{\raise0.7ex\hbox{$1$} \!\mathord{\left/ {\vphantom {1 2}}\right.\kern-\nulldelimiterspace} \!\lower0.7ex\hbox{$2$}}}} \left( \frac{E}{H} \right)^{{{\raise0.7ex\hbox{$2$} \!\mathord{\left/ {\vphantom {2 3}}\right.\kern-\nulldelimiterspace} \!\lower0.7ex\hbox{$3$}}}} \frac{P}{{c^{3/2} }} $$

where *K*_*c*_ is fracture toughness, *a* is indent impression radius from the center of the impression to the corner, *l* is surface crack length, *E* is Young’s modulus, *H* is hardness, *P* is maximum indenter load, and *c* is the length from the center of the indent impression to the crack tip, i.e., *a + l*, and *x*_*v*_ is a fitting factor. This equation is valid for the Palmqvist radial crack system. For low-load nanoindentation, a fitting factor of 0.022 was calibrated by Cuadrado et al. and is used here.^24^

EBSD experiments were carried out using a Zeiss Merlin FEG-SEM with a Bruker Quantax e-flash detector controlled using Bruker Esprit 2.1 software. The microscope voltage was 20 kV and the beam current 20 nA, with an acquisition time of 50 ms per pixel, and a 100 nm pixel step size. Diffraction patterns were acquired with a resolution of 800 × 600 pixels and were analyzed using the XEBSD code developed at the Department of Materials, University of Oxford, and Imperial College London.^36^–^38^

High-angular-resolution EBSD analysis is explained in detail in Refs.^39^,^40^, but is summarized here. Diffraction patterns acquired at each pixel are separated into 40 partially overlapping regions of interest (ROI). Each ROI undergoes a fast Fourier transform and is cross-correlated to the corresponding ROI in a nominally unstrained reference diffraction pattern, with the same crystal orientation. The translation vector between matching ROIs in the reference diffraction pattern and the current pixel diffraction pattern is calculated. A self-consistent deformation tensor for the diffraction pattern is calculated, mathematically representing movement of Kikuchi bands in the EBSD pattern.^41^ Cooperative movement of Kikuchi bands is caused by crystal rotations, while changes to Kikuchi bands relative to each other are caused by changes to interplanar spacings from an applied deviatoric strain. Elastic stresses are calculated from elastic strains using the anisotropic Hooke’s law with elastic constants from the Materials Project database.^42^ Assuming surface traction-free plane stress, the final *ε*_33_ strain component can be calculated.^41^

HR-EBSD analysis directly accounts for elastic strain and stress. Plastic deformation by dislocations is assessed indirectly based on the lattice deformation around dislocations. Dislocations contribute to a net lattice curvature due to the extra half-planes of atoms. The spatial gradient of lattice rotations measured by HR-EBSD can be related to the density of geometrically necessary dislocations (GND) required to cause the measured lattice curvature.^43^ The measured lattice curvature is a net effect of dislocations in the structure, not necessarily dislocations which have contributed to plastic deformation, and is a lower bound as dislocations of opposite sign will cancel out their effect on lattice curvature within a diffracting interaction volume.^39^ This derivation of curvature caused by a net density of dislocations can extend into three dimensions as a tensor that can be solved based on lattice rotations and elastic strains measured using HR-EBSD.^41^,^43^ With the angular and strain sensitivity of cross-correlation HR-EBSD, sensitivity in GND density maps is ~10^12^ m^−2^.^41^

Raman spectroscopy experiments used a Witec Alpha 300R confocal Raman microscope in the Materials Research Facility at UKAEA. A green 532 nm laser operating at 10 mW power was used to acquire spectra through a 100× objective lens with 0.5 s integration time. The 50-µm-diameter optical fiber connecting the microscope to the spectrometer acted as a confocal aperture, achieving a depth resolution ~1 µm. The sample was scanned to produce a two-dimensional map of the indentation impression with step size of 200 nm. Spectra were acquired for each pixel in the map with curves fit using a Lorentz function in Witec Project 5 software. The position of the SiC transverse optic peak (~789 cm^−1^) was extracted to form maps of Raman peak position shift, Δ*ω*, relative to a nominally unstressed position in the same sample. An indented sample of single-crystal silicon (001) was similarly investigated using the longitudinal optic peak at ~520 cm^−1^.

Stress is linearly related to the change in Raman peak position by *σ* = Δ*ω* × *R*, where *R* is a piezospectroscopic coefficient. This relationship depends on stress state, where for hydrostatic stress, $\sigma = \frac{\Delta \omega \times R}{3}$, and for balanced biaxial stress $\sigma = \frac{\Delta \omega \times R}{2}$. For 6H-SiC, *R* = −849.9 MPa cm from DiGregorio and Furtak^44^ is used. This coefficient was calibrated using hydrostatic pressure $\left( {\frac{R}{3} = - 283.3\;{\text{MPa}}.{\text{cm}}} \right)$ but is considered by the authors of Ref. 44 to be a coefficient for average stress across all crystal directions and so could be applied to other stress states including biaxial. Raman stress mapping in silicon has been extensively studied, and the coefficients have been well characterized theoretically and experimentally.^45^–^47^. The sign of the coefficient indicates that a shift to higher wavenumber corresponds to a compressive residual stress, while a tensile stress state causes a shift to lower wavenumber.

To investigate subsurface fracture, indents were cross-sectioned using a Zeiss Auriga FIB-SEM. A coarse trench was milled away from the indent with 30 kV, 4 nA Ga^+^ ions to reveal a subsurface cross-section to the SEM field of view, then fine slices (~30 nm thick) were milled at 30 kV, 240 pA with automated image acquisition after every three slices.

## Results

### Indentation Fracture Toughness

**Fig. 1:**
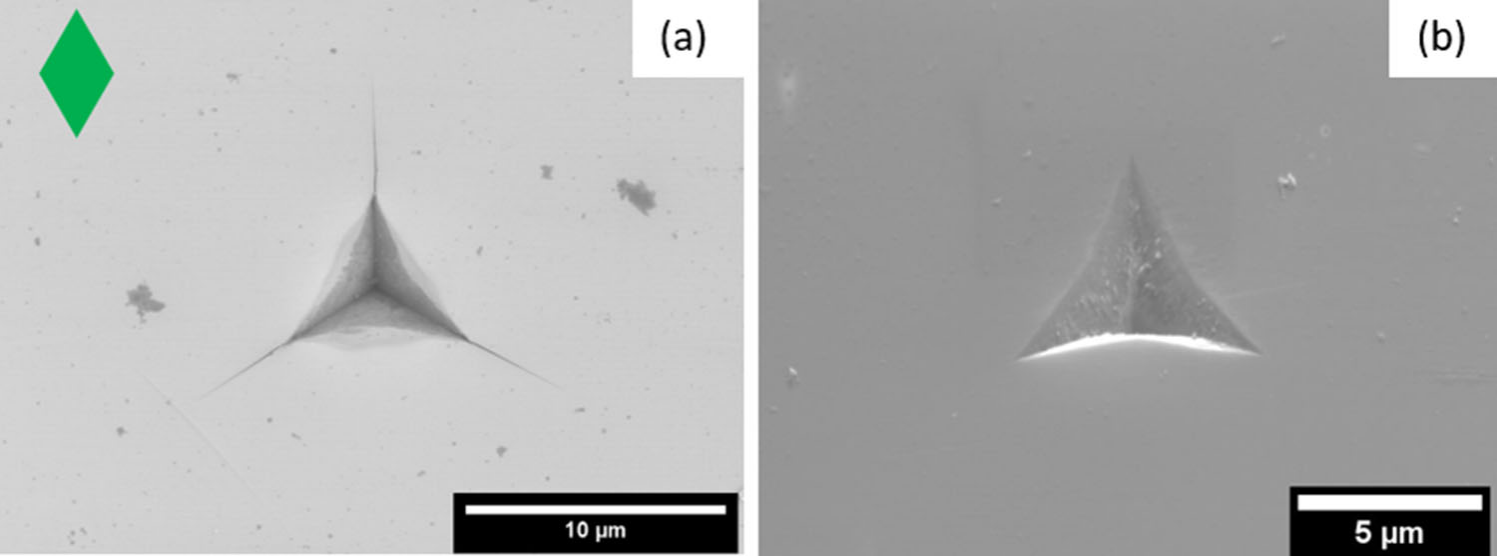
(a) SEM micrograph of a 1 µm Berkovich indent in 6H-SiC with the crystallographic unit cell orientation represented by the green diamond. (b) 1 µm Berkovich indent in irradiated 6H-SiC at the same crystallographic orientation as (a). No radial cracks.

Indentations in unirradiated SiC show radial surface cracks emanating from the corners of the indent impression (Fig. 1a). These radial surface cracks were 4.3 ± 0.1 µm long, corresponding to an indentation fracture toughness of 2.5 MPa√m assuming Palmqvist radial cracking under these low-load indentations. With no radial cracks in the irradiated 6H-SiC (Fig. 1b), no indentation fracture toughness can be calculated using this method.

### Stress Distributions

**Fig. 2:**
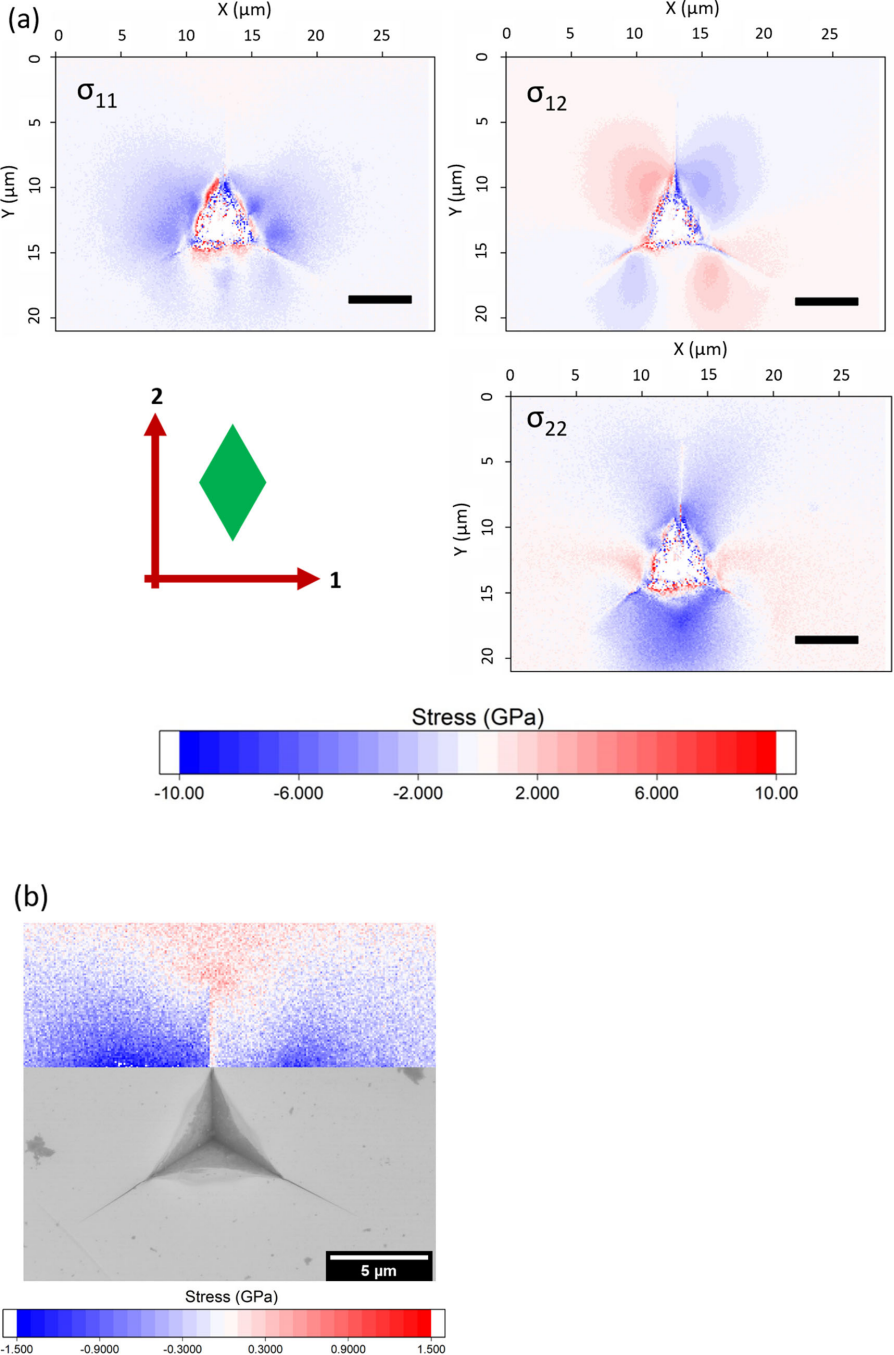
(a) HR-EBSD plane stress tensor of a 1 µm Berkovich indent in 6H-SiC. Scale bar is 5 µm. Axes and crystal unit cell orientation are shown in the lower left. (b) The *σ*_11_ component shown on a magnified stress scale.

HR-EBSD maps of the components of the plane stress tensor around an indent in unirradiated SiC are shown in Fig. 2. This displays the residual stresses around the indent impression, showing large compressive radial stresses, shown most clearly as the *σ*_22_ component below the indentation. The hoop stresses (e.g., the *σ*_11_ component below the indentation) are relatively small. The tensile stresses ahead of the crack tip reach 600 MPa in the *σ*_11_ component, as shown more clearly on a magnified scale in Fig. 2b.

**Fig. 3:**
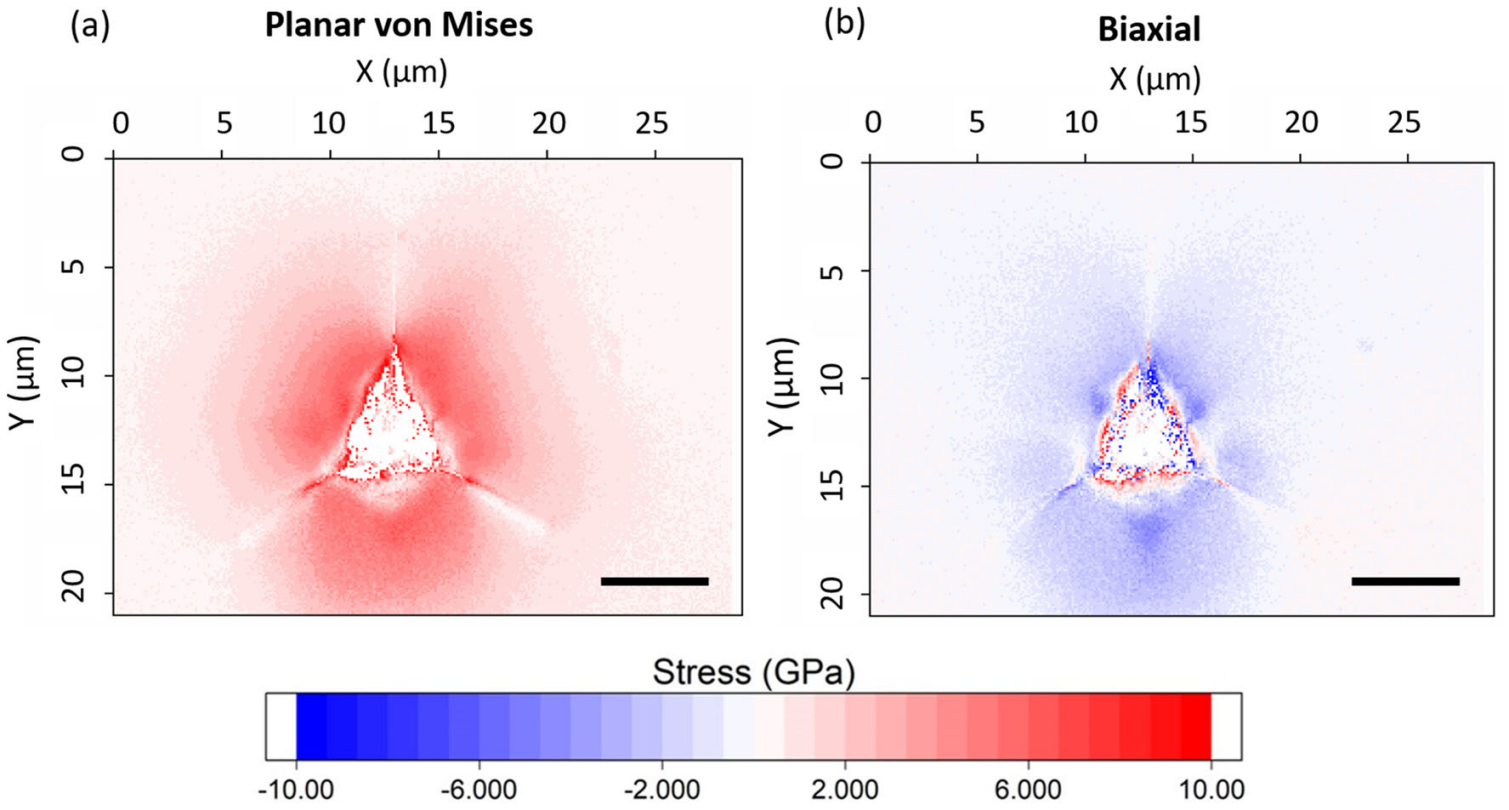
(a) Planar von Mises and (b) biaxial stress maps of a 1 µm Berkovich indent in 6H-SiC. Scale bar is 5 µm. The biaxial stress is the two-dimensional analog of the hydrostatic stress component in three dimensions.

The total elastic deformation around this indentation can be seen in the planar von Mises stress and biaxial stress maps in Fig. 3. There is stress relief along the cracks, and a lower residual stress near the indent impression.

**Fig. 4:**
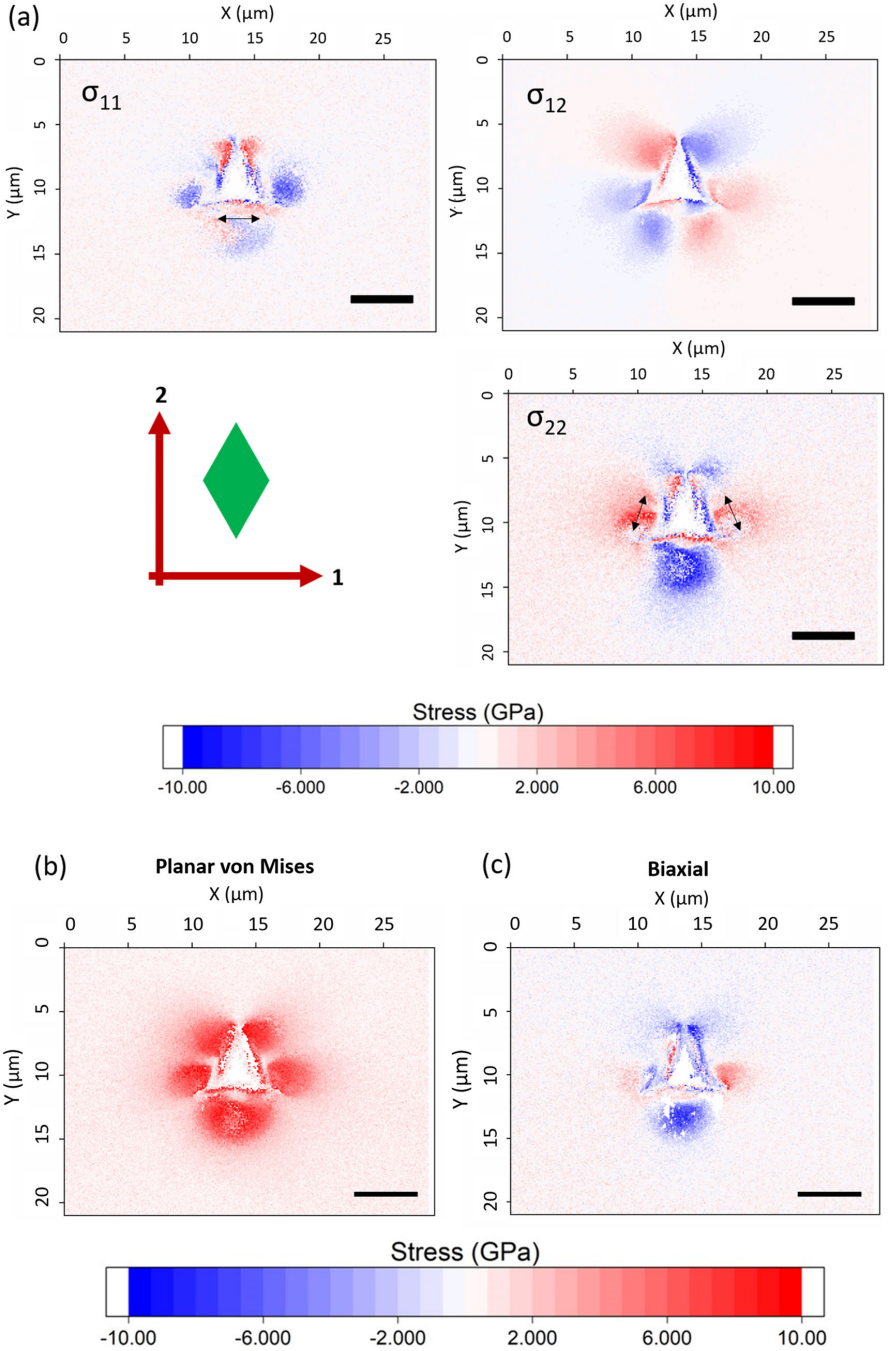
(a) HR-EBSD plane stress tensor of a 1 µm Berkovich indent in irradiated 6H-SiC. Scale bar is 5 µm. Axes and crystal unit cell orientation are shown in the lower left. Arrows indicate direction of hoop stress. (b) Planar von Mises and (c) biaxial stress maps for the same indent. Stresses are relative to stress far from the indent, which is shown to be compressive.

Indent impressions in the irradiated region of the specimen have significantly different elastic deformation, shown in Fig. 4. These HR-EBSD maps use a reference diffraction pattern away from the indent, but within the irradiated region. This is *not* a stress-free position, but has been shown to have a compressive biaxial stress calculated to be −1.9 GPa using HR-EBSD (larger with other measurement techniques).^13^ These stress maps are calculated and plotted without this compressive biaxial stress. Elastic deformation is localized closer to the indent than in the unirradiated case, and reaches a relatively higher magnitude. Additionally, there is a tensile hoop stress (relative to the regions far from the indentation) around the indent impression, which was not present in the unirradiated indents. This is seen most clearly in the *σ*_22_ component adjacent to the sides of the indentation, and in the *σ*_11_ component below the lower face of the indentation, indicated with arrows in Fig. 4a.

**Fig. 5:**
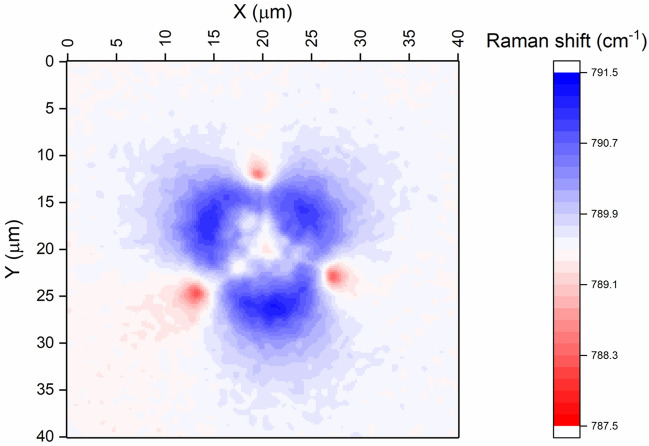
Raman piezospectroscopic map of a 1 µm Berkovich indent in 6H-SiC. The color scale is centered on 789.5 cm^−1^, the position of the 6H-SiC TO peak away from the indent in nominally unstressed material. Higher wavenumbers are compressive stresses, mapped in blue, while lower wavenumbers are tensile, mapped in red.

Raman piezospectroscopic mapping of the unirradiated indent (Fig. 5) shows a qualitatively similar spatial variation of residual elastic stress as the HR-EBSD biaxial stress maps shown above. Stress relief is observed as a smaller Raman shift (lighter blue in Fig. 5) following the cracks to the crack tips where there is a tensile stress shown in red. A lower compressive stress is seen close to the indent impression, possibly caused by relaxation from plastic deformation, pile-up, or subsurface fracture. The peak shift at the crack tips is −1.3 cm^−1^ corresponding to a uniaxial tensile stress of 368 MPa. The maximum peak shift in the compressive regions is +1.8 cm^−1^. Assuming a hydrostatic stress state, *σ* = −510 MPa. If DiGregorio and Furtak are correct in claiming that their piezospectroscopic coefficient is valid in nonhydrostatic conditions, an assumed biaxial stress would be −765 MPa and a uniaxial (*σ*_22_) stress −1.53 GPa.^44^ The stresses measured by Raman spectroscopy are considerably lower than those measured by HR-EBSD. This is probably due to the larger interaction volume in this optically transparent single crystal incorporating some less strained material beneath the surface, despite the confocal aperture. Guo and Todd showed the effect of depth resolution on observed residual stress around micro indents in alumina.^48^ Errors of greater than 40% could arise when non-confocal (~14 µm depth resolution) fluorescence mapping was used compared with confocal (~3 µm depth resolution in their experiments). The difference in residual stress measured by HR-EBSD and Raman spectroscopy is related to the depth resolution (~40 nm in EBSD, ~1 µm in Raman), and the fact that residual stress around indents falls steeply below the specimen surface, exacerbated by the small sizes of the indents in this work.

The Raman spectrum of irradiated SiC is dominated by radiation defects, which prevents this technique from being used for accurately mapping residual stresses around indents.^49^

### Plastic Deformation

**Fig. 6:**
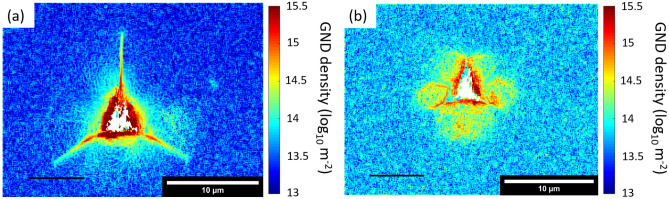
GND density maps showing where plastic deformation occurred around (a) unirradiated indent and (b) irradiated indent.

Geometrically necessary dislocation (GND) density maps of unirradiated and irradiated indents (Fig. 6) show clear differences in the residual plastic deformation. Without irradiation, residual plastic deformation is localized near the indent impression, approximately in the region of lower residual elastic deformation observed in the elastic stress maps in Figs. 3 and 4. Cracks can clearly be seen as discontinuities in lattice curvature. The area of high GND density is in an approximate circle connecting the corners of the indent impression. In contrast, the region of high GND density in the irradiated material extends further from the indent, approximately in the same shape as the elastic deformation. The GND density is also higher in the background region of the irradiated material. This is considered to be a physical effect of radiation damage, where radiation defects cause lattice curvature and a calculated GND density. This highlights the point that a region of high GND density does not necessarily mean it is a region of high plastic deformation; however, in this case, GNDs near the indent are a clear sign of plastic deformation. Transmission electron microscopy (TEM) analysis around indentations in SiC shows basal plane and prismatic dislocations at room temperature.^50^,^51^

### Crack Growth and Residual Stress State Modification

**Fig. 7:**
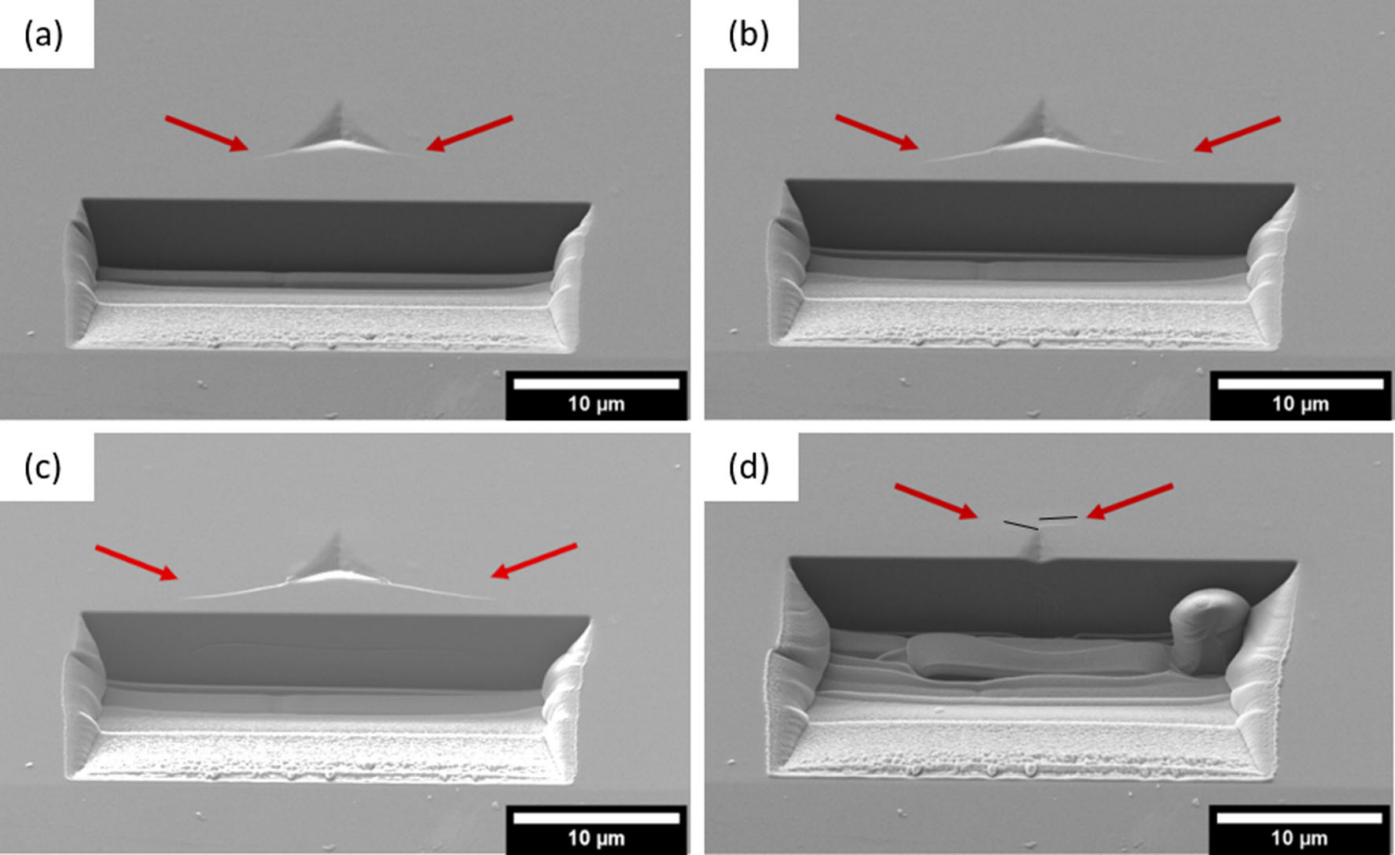
FIB slicing of an indent in irradiated 6H-SiC showing: (a) radial cracks growing from the lower corners of the indent impression as material is FIBed away, (b) the slice immediately before cracks jump forward and (c) the slice immediately after, and (d) two cracks growing from the top corner of the indent impression. The black lines are placed slightly above and parallel to the length of the cracks to show their path.

An irradiated indent was cross-sectioned to see whether there was subsurface fracture. During FIB milling, radial cracks grew stably from the indentation corners closest to the trench to a length of ~4 µm as material was removed (Fig. 7a and b). In the subsequent FIB slice (Fig. 7c), the cracks suddenly elongated considerably. A subsurface lateral crack can be seen to have developed simultaneously. No crack is visible from the upper indent corner until later in the slicing process (Fig. 7d). Instead of a single radial crack, two tangential cracks grow, near parallel to the plane of FIB slicing and stress relief. A video of the whole cross-section imaging process can be seen in the Electronic Supplementary Material.

**Fig. 8:**
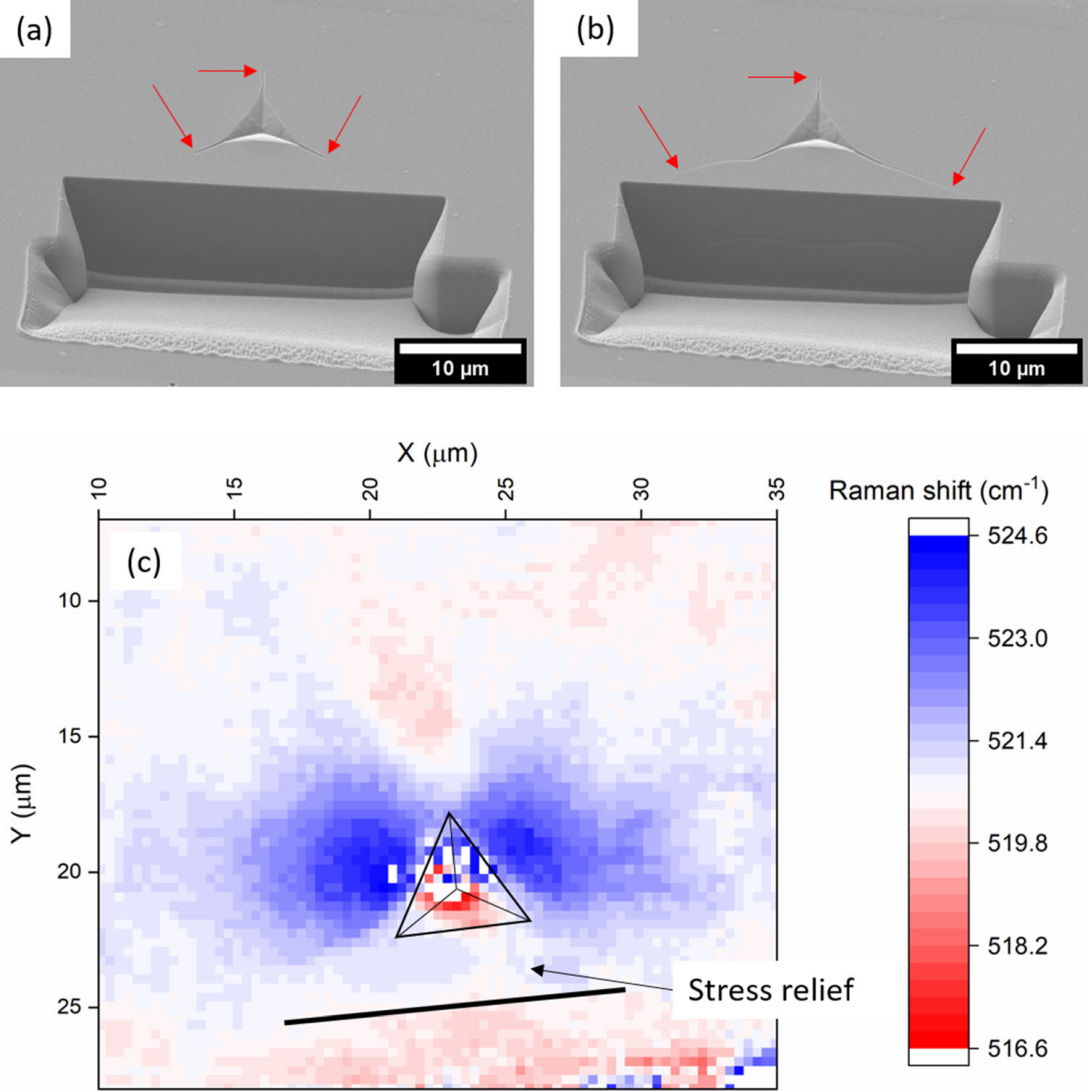
(a), (b) Crack growth during FIB cross-sectioning of a 1 µm Berkovich indent in silicon (001) surface. Red arrows point at crack tips. Slicing was stopped immediately after crack growth, and residual was mapped on the surface using Raman spectroscopy (c). The indent impression is indicated with a triangle, and the solid line marks the position of the edge of the FIB trench.

A crack “jump” similar to that in Fig. 7c is seen during cross-sectioning of indents in unirradiated SiC, and silicon, but without the stable crack growth beforehand. The process of FIB imaging during alignment amorphizes the surface of SiC, preventing stress mapping using Raman or HR-EBSD methods after FIB cross-sectioning. Silicon, however, is more resistant to this low-dose FIB damage and retains a crystalline Raman signal. A 1 µm Berkovich indent was made in a sample of unirradiated single-crystal silicon (001) cleaved from a larger semiconductor substrate wafer which had been polished on one side. This indentation was cross-sectioned, with the sectioning stopped as soon as the crack jump was observed. The surface of this sample was mapped using Raman spectroscopy, with the zero-stress peak position being taken in the same specimen far from the indent impression and FIB-damaged zone (Fig. 8). Similar features to the Raman map of the unirradiated SiC indent (Fig. 5) can be seen with tensile stress ahead of the upper crack tip and compressive “lobes” near the indent faces. The region closest to the cross-sectioning trench, between the elongated cracks, does not have this compressive stress.

## Discussion

### Indentation Fracture and Apparent Toughness

The surface of this neon-implanted silicon carbide sample has already been shown to contain a biaxial compressive stress of several GPa caused by constrained swelling, which contributes to large hardening.^13^ A consequence of this compressive stress is the prevention of crack growth, making the irradiated specimen appear tougher. A sample of SiC irradiated at 750°C which received less radiation damage (and lower swelling and hardening as a result) had radial indentation cracks, but shorter than in the unirradiated case.^52^ This observation agrees with the results from Jiang et al. where indentation cracks became shorter, giving an apparent increase in indentation toughness with increasing ion implantation damage and swelling.^23^

At the surface, where there is the lowest damage at less than 0.8 dpa, there is also the smallest swelling-induced compressive residual stress. Nevertheless, this is still sufficient to prevent indentation fracture in this work. Due to damage gradients in ion implantation, it is possible that the residual stress may be low enough to allow the formation of indentation cracks near the surface, which are suppressed below the surface. This situation is more complex, as assumptions of subsurface crack morphology for calculating fracture toughness are invalid. Jiang et al. report a 70% increase in apparent fracture toughness at 0.2 peak dpa (~0.067 surface dpa), suggesting that very small damage doses are required to cause significant crack shortening.^23^ Their indents were deeper than the damaged layer, while in this work, with 1 µm indents in a 1.2 µm damaged layer, the entire indent impression experiences a large compressive biaxial stress state, opposing fracture.

The process of FIB cross-sectioning the irradiated indent locally relieves the biaxial radiation-induced stress as shown in Fig. 8. As this compressive stress is removed, the cracks undergo stable crack growth due to the tensile hoop stress, growing to a crack length close to the unirradiated case. This suggests that the actual fracture toughness of the material has not been significantly changed by radiation defects, confirming that crack suppression is due to the compressive residual stress caused by the substrate. As the biaxial residual stress is only relieved near one face of the indent impression, the opposite radial crack does not grow and the residual stress is not symmetric; therefore, these crack lengths should not be used for fracture toughness calculations. Swelling is unconstrained in free-standing neutron-irradiated specimens, so there is no compressive residual stress on the surface and fracture toughness is not significantly altered by radiation defects, agreeing with this interpretation for ion-implanted specimens.^15^, ^16^

The final FIB cross-sectioning shows radial median cracks meeting beneath the indent impression; however, it is unclear whether these cracks began as median cracks or were initially Palmqvist type and grow to median cracks during the crack jump. Additionally, this procedure shows the appearance of lateral cracks during cross-sectioning (Fig. 7c); they were not present before sectioning. Indentation fracture toughness equations require an assumption of subsurface crack morphology, which is unclear here.^24^,^34^ A better technique may be conventional top-down incremental polishing where lateral constraint is not removed.

### Effect of Ion Implantation on Plastic Deformation and Hardness

As well as changing fracture properties, ion implantation also changes the elastic and plastic deformation characteristics of indentation. Ion implantation causes a 12% to 15% increase in hardness and a peak reduction in elastic modulus of 12% as measured by nanoindentation in the samples in this work.^13^ The reduction in elastic modulus results from disruption of the crystal lattice, but the effect on hardness has several contributions. Part of this is related to the manner in which the volume expansion associated with the indentation is accommodated when the elastic limit is exceeded. In unirradiated material, radial cracks accommodate the indentation expansion by relieving the hoop stress, limiting plastic deformation to the region in the immediate vicinity of the indentation as observed here. The suppression of fracture by the surface compressive stress in the irradiated specimen requires the expansion of the indentation to be completely accommodated by plastic deformation beyond the elastic limit, pushing the plastically deformed region further out from the indentation, as seen in Fig. 6. In addition, the compressive biaxial stress caused by constrained swelling acts to oppose the expansion associated with the indentation, making the specimen appear harder. This is the principle by which indentation can be used to measure biaxial residual stresses.^53^, ^54^

Other materials susceptible to radiation swelling are likely to have compressive residual stresses in ion-implanted layers, which could change indentation deformation and the measurement of radiation-induced changes to mechanical properties. In helium-implanted tungsten, lattice swelling alone causes a compressive biaxial stress of −490 MPa, which is likely to be larger if total swelling is considered.^55^ Deformation around spherical indents was altered, partially due to the swelling stress, and partially due to radiation defects.^17^,^18^ Residual stress from radiation swelling in implanted SiC in this work, using a sharp indenter, causes a more significant change to deformation with the suppression of fracture; this effect should not be neglected when studying ion-implanted metals.

## Conclusion

Ion irradiation significantly alters indentation deformation in SiC. This radiation damage appears to improve fracture toughness; however, this is an artificial consequence of compressive residual stresses caused by constrained radiation swelling. The true fracture toughness of the material is unclear from nanoindentation techniques but does not appear to have been significantly altered by radiation defects. This compressive biaxial stress, combined with suppressed fracture, also modified plastic deformation around indentations and contributed to the large increase in hardness of ion-implanted SiC.

These results suggest that the deformation of ion-irradiated SiC during nanoindentation is fundamentally different from deformation of neutron-irradiated SiC. Ion irradiation with these conditions does not appear to be suitable as a surrogate for replicating neutron irradiation damage. Ion irradiation conditions could be adjusted to avoid swelling-induced stresses, for example, in thin films where ions pass through such that there is no substrate effect. Care should be taken in interpreting mechanical properties of ion-irradiated materials measured by nanoindentation.

The in-plane residual stress tensor around indents in fractured, and nonfractured SiC has been measured using HR-EBSD, providing insights into the interplay of residual stress state, crack formation, and plastic deformation. The process of FIB cross-sectioning alters the stress state and changes crack morphology, which makes observing the subsurface crack structure of these small indents challenging.

## Supplementary Information

Below is the link to the electronic supplementary material.

Supplementary file1 (AVI 11708 kb)
